# Antimicrobial Peptides as Mediators of Innate Immunity in Teleosts

**DOI:** 10.3390/biology4040607

**Published:** 2015-09-25

**Authors:** Barbara A. Katzenback

**Affiliations:** Department of Biology, University of Waterloo, 200 University Ave West, Waterloo, ON N2L 3G1, Canada; E-Mail: barb.katzenback@uwaterloo.ca; Tel.: +1-519-888-4567

**Keywords:** antimicrobial peptides, innate immunity, teleosts, fish, aquaculture, piscidins, defensins, hepcidins, cathelicidins, NF-κB

## Abstract

Antimicrobial peptides (AMPs) have been identified throughout the metazoa suggesting their evolutionarily conserved nature and their presence in teleosts is no exception. AMPs are short (18–46 amino acids), usually cationic, amphipathic peptides. While AMPs are diverse in amino acid sequence, with no two AMPs being identical, they collectively appear to have conserved functions in the innate immunity of animals towards the pathogens they encounter in their environment. Fish AMPs are upregulated in response to pathogens and appear to have direct broad-spectrum antimicrobial activity towards both human and fish pathogens. However, an emerging role for AMPs as immunomodulatory molecules has become apparent—the ability of AMPs to activate the innate immune system sheds light onto the multifaceted capacity of these small peptides to combat pathogens through direct and indirect means. Herein, this review focuses on the role of teleost AMPs as modulators of the innate immune system and their regulation in response to pathogens or other exogenous molecules. The capacity to regulate AMP expression by exogenous factors may prove useful in modulating AMP expression in fish to prevent disease, particularly in aquaculture settings where crowded conditions and environmental stress pre-dispose these fish to infection.

## 1. Introduction

Antimicrobial peptides (AMPs) are a diverse group of individually unique small peptides (18–46 amino acids) found in plants to animals that display broad-spectrum antimicrobial activity towards bacteria, viruses, parasites, and fungi. Recent studies have also implicated AMPs as host defense peptides (HDPs) that function as immunomodulators adding a layer of complexity to their functional role in defense against pathogens. AMPs can be produced at a constitutive level but can also be induced in response to pathogens. The major pathway of AMP production is through the recognition of pathogen associated molecular patterns (PAMPs) via Toll-like receptors (TLRs) thereby triggering an intracellular signaling cascade involving MyD88, TRAF6, IRAK1 and IKK [1,2]. This signal transduction pathway ultimately leads to the activation and translocation of NF-κB to the nucleus where it transcriptionally activates AMPs and other pro-inflammatory molecules [1,2]. The AMPs are synthesized in inactive forms as part of a prepropeptide and are enzymatically cleaved to release the functional mature peptide. The widespread nature of AMPs suggests that they are an evolutionarily ancient mechanism of host defense against pathogens and their diversity in amino acid sequence and structure points to an evolutionary pressure to cope with a specific milieu of microbes. The > 1000 AMPs identified to date has necessitated their organization in databases, such as the Antimicrobial Peptide Database [3].

Despite the reports of AMPs in amphibians and humans in the late 1970s and early 1980s, the identification and study of fish AMPs only began in the mid to late 1990s. To date, over 90 fish AMPs have been identified [3] and fall into five major families based on their structure; β-defensins, cathelicidins, hepcidins, histone-derived peptides, and the fish specific piscidins. These fish AMPs appear to retain a similar overall structure, broad-spectrum antimicrobial activity and immunomodulatory function to that of other vertebrate AMPs. The focus of this review is to explore the regulation of teleost AMPs in response to pathogen challenge and the immunomodulatory function of teleost AMPs studied thus far. A comprehensive review of the antimicrobial activity of fish AMPs towards a wide array of fish and human pathogens and the minimal inhibitory concentrations at which antimicrobial activity occurred are reviewed in [4] and will not be discussed herein. The persistence of AMPs in nature for over millions of years demonstrates the inability of microbes to develop resistance against AMPs, making AMPs an interesting target for use as antibiotic replacements in the medical field and in aquaculture as therapeutics.

## 2. β-Defensins

### 2.1. Gene and Protein Structure, Expression and Function

Fish defensins, most closely resembling that of mammalian β-defensins, were first identified in zebrafish (*Danio rerio*), fugu (*Takifugu rubripes*) and tetraodon (*Tetraodon nigroviridis*) [5]. Differing β-defensin gene copy numbers are present in fish; one gene copy has been identified in gilthead seabream (*Sparus aurata*) [6], orange-spotted grouper (*Epinephelus coioides*) [7], Atlantic cod (*Gadus morhua*) [8], Nile tilapia (*Oreochromis niloticus*) [9], Chinese loach (*Paramisgurnus dabryanus*) [10] and medaka (*Oryzias latipes*) [11]; two in blunt snout bream (*Megalobrama amblycephala*) [12]; three in zebrafish (*Danio rerio*) [8] and carp (*Cyprinus carpio* L.) [13,14]; four in rainbow trout (*Onchorhynchus mykiss*) [15,16]; and five in olive flounder (*Paralichthys olivaceus*) [17]. Collectively, the β-defensin genes have a three-exon/two-intron structure [5,8,9,11,16,18,19]. In fish with multiple β-defensin gene copies, some gene copies were linked and present on the same chromosome while other β-defensin genes were found on a separate chromosome [5], providing evidence for multiple defensin loci in fish. 

The β-defensins of fish appear to be produced as a prepeptide. The prepeptide is synthesized as a precursor that is comprised of a 18–24 amino acid signal peptide and a 39–45 amino acid mature peptide, with the exception of olive flounder in which a 15 amino acid proregion is also present, and is cleaved to produce the mature peptide [5,15,16,17]. The resulting mature β-defensin peptides are small (4–6 kDa), share the 6 conserved cysteine residues present in the vertebrate defensins, retain the CPRRYK/R motif between cysteines 4 and 5 and have an overall net cationic charge between +1 and +7 [5,6,10,11,15,16,18]. Fish β-defensins appear to fall into two main groups and have an overall three beta-strand secondary structure, although some β-defensins are predicted to possess an extra alpha-helix at the N-terminus of the three-beta strand structure [5,8].

During embryonic development β-defensin transcripts are expressed early on and at constitutive levels [8,12,17] suggesting that β-defensin may play a key role in host defense during embryogenesis. In adult fish, the expression pattern and levels of β-defensins varies greatly depending on the fish species and the defensin gene [12]. However, the primary expression of β-defensin appears to be in the immune and mucosal tissues including the intestine, gill, kidney, spleen, skin, and liver [6,13,14,18,19,20], with expression also detected in the muscle, stomach, eye, gonad, heart, blood, monocytes and lymphocytes [5,6,11,15,16,20,21]. In some fish species, β-defensin transcripts were also detected in the pituitary, testis [7], swim bladder and peritoneum (with low levels in the skin and kidney) [8]. However, β-defensin expression is seemingly absent in the brain [16]. The broad distribution of β-defensin in fish tissues suggests that β-defensin plays a role in defense against infection in these tissues or may play broader role in fish physiology. 

In fish model systems where antibodies to species-specific β-defensin have been generated, it appears that mRNA levels of β-defensins correspond to protein levels [7,11,19]. Whether this is true for all fish species or just in the fish species examined in the aforementioned study remains to be determined. However, the confirmation of mRNA transcripts to protein correlation suggests that mRNA expression is likely a good indicator of β-defensin protein production. One study noted the translocation of β-defensin peptides from the cytoplasm to the nucleus where viral replication was occurring in infected cells [18], demonstrating that β-defensins are actively mobilized to sites of pathogen replication within a cell. Recombinant or synthetic fish defensins have demonstrated antimicrobial activity towards Gram-negative bacteria [6,11,17,19,20], Gram-positive bacteria, [19,20] and viruses [8,15,18], although the antimicrobial activity was dependent on the fish species and the defensin gene. A complete list of MICs for fish β-defensins and the pathogens they have been tested against can be found in [4]. Interestingly, fish β-defensins have not been tested against fungi or parasites and represents an area of study that requires further investigation. 

### 2.2. Regulation of Defensins by Pathogens/Exogenous Factors

The expression of β-defensins is modulated in response to a number of pathogens (Table 1, Table 2, Table 3 and Table 4), similar to that observed for other teleost AMPs. Inducers of β-defensin transcript levels include pathogen components such as LPS [10], bacterial DNA [11], unmethylated CpG oligodeoxynucleotides [11] and β-glucan [13], as well as pathogens including Gram-negative bacteria, Gram-positive bacteria and viruses (see Table 1, Table 2, Table 3 and Table 4 and references therein). Pathogen challenge usually results in the rapid induction of β-defensin transcript levels 12 h post challenge, with transcript levels decreasing thereafter [10,18], suggesting that β-defensin, like other AMPs, are important molecules in the first line of defense against pathogens. The regulation of β-defensin transcripts in tissues in response to pathogen challenge is species and gene specific, although upregulation of β-defensin is commonly observed in the head kidney, spleen, liver, skin, gills and eye [6,8,11,13,17,18]. However, in one study in which common carp were infected with carp herpes virus 3, a decrease in the β-defensin skin transcript levels was observed suggesting that this particular virus may be able to immunomodulate the host to create an immunosuppressed environment permissive to viral replication [22]. The constituents of a fish’s diet can also act to regulate β-defensin expression. Gilthead seabream fed microalgae diets exhibited an increase in the expressions of defensin transcripts in the head kidney [23], while rainbow trout fed peptidoglycan spiked food showed an increase in defensin mRNA levels in the skin and gill tissues [24]. These studies suggest that pathogens, their components and entities within fish feed can modulate the expression of AMPs and may impact the susceptibility or resistance of fish to disease.

Examination of the upstream promoter region of β-defensin genes has revealed binding sites for myocyte-specific enhancer factor-2 (MEF-2), activating protein-1 (AP-1), CCAAT-binding factor (CBF), interferon regulatory factor (IRF), CCAAT/enhancer binding protein (C/EBP), IL-6 responsive element binding protein (IL-6 RE-BP), stimulating protein-1 (Sp1), lymphocyte-enhancer factor-1 (LEF-1), nuclear factor of activated T-cells (NF-AT) [17,19] and NF-κB [11] transcription factors. The prediction of a multitude of transcription factor binding sites suggests that β-defensins are regulated by a number of upstream activation pathways leading to transcriptional activation of β-defensins in fish in response to varied stimuli, supported by the array of pathogen components and pathogens that can modulate the expression of β-defensins in fish. 

### 2.3. Modulation of Immunity by Defensins

Studies in fish suggest that β-defensins may also act as HDPs and are capable of modulating the immune response. *In vitro* studies have demonstrated that treatment of rainbow trout RTG-2 cells with rainbow trout defensin [9] or ZF4 zebrafish embryonic fibroblast cells with zebrafish β-defensin-2 (zfBD2) resulted in the upregulation of Mx gene expression [25], suggestive of a type I interferon response. *In vivo* studies in fish have confirmed the increased mRNA levels of Mx and pro-inflammatory cytokines such as IL-1β and TNF-α in fish tissues in response to injection with β-defensin [18].

Furthermore, expression of zfBD2 in zebrafish ZF4 cells promoted the translocation of NF-κB from the cytoplasm to the nucleus thereby linking NF-κB pathway activation, and possibly upstream TLR signaling, as a mechanism of immune cell activation in response to β-defensins [26]. The activation of NF-κB coincides with the increased transcript levels of pro-inflammatory cytokines observed in cells and tissues upon treatment with β-defensins as NF-κB is known to transcriptionally activate AMPs and a number of pro-inflammatory molecules. Functional *in vitro* studies have shown defensins to be chemoattractive to gilthead seabream head kidney leukocytes [6] and to stimulate the phagocytic activity of Atlantic cod head kidney leukocytes [8], while *in vivo* studies with zebrafish suggest a recruitment of fish cytotoxic cells to the site of β-defensin injection [25]. It is perhaps through some or all of these immunostimulatory mechanisms that the *in vivo* expression of zebrafish BD2 conferred a partial enhancement of resistance towards spring viraemia of carp virus (SVCV) infection compared to wildtype zebrafish. 

## 3. Cathelicidins

### 3.1. Gene and Protein Structure, Expression and Function

Teleost cathelicidins were first identified in rainbow trout [27]. Since then, varying numbers of cathelicidin genes have been identified in different fish species; one in Arctic charr (*Salvelinus alpinus*) [24], brown trout (*Salmo trutta fario*) [24,25], ayu (*Plecoglossus altivelis*) [28], grayling (*Thymallus thymallus*) [25], thick-lipped lenok (*Brachymystax lenok*) [29], Chinook salmon (*Onchorhynchus tshawytscha*) [30]; two in Atlantic salmon (*Salmo salar*) [31,32], brook trout (*Salvelinus fontinalis*) [25]; three in in Atlantic cod (*Gadus morhua*) [24]; and four in rainbow trout (*Oncorhynchus mykiss*) [27,32,33]. In species that have multiple cathelicidin genes, such as rainbow trout, the multiple gene copies are found on different chromosomes [33] and are believed to have arisen as the result of whole genome duplication. Teleost cathelicidins fall into two classes—cathelicidin 1 and cathelicidin 2, distinguished by the presence and absence of cysteines and the formation of disulphide bonds, respectfully [32]. Little homology exists between the classes of cathelicidins. In the majority of fish species that have a single cathelicidin gene, they appear to have cathelicidin 1 type peptides. However, in Chinook salmon (*Onchorhynchus tshawytscha*) only one cathelicidin gene was found and closely grouped with the cathelicidin 2 class [30].

Amongst fish species, the majority of the cathelicidins have a four-exon/three-intron genomic structure like that of mammalian cathelicidins [24,27]. The cathelicidin transcripts encode for a 159–222 amino acid polypeptide [24,25,27,32] composed of a signal sequence, a N-terminal preproregion which contains the highly conserved cathelin domain, encoded by exons 1–3, and a highly variable C-terminal sequence, encoded by exon 4, that contains a QKIRTRR elastase cleavage site and a unique cathelicidin mature peptide [24,25,27,28,32]. The sequence of the fourth exon amongst fish is hypervariable and gives rise to the suite of distinct mature peptides that differs between and within fish species [25]. The mature peptide is 47-69 amino acids, has a charge of +8 to +17 [24,25,27,30] and studies on rainbow trout cathelicidins suggest that fish cathelicidins are mainly β-sheet and random coil structures [33]. Variations to the general structure of cathelicidins have been discovered. In Arctic charr (*Salvelinus alpinus*) and brook trout (*Salmo trutta fario*) the identified cathelicidins had a deletion of exon 3 [24]. In rainbow trout, one of the four identified cathelicidin genes was missing exons 1, 2, and 3—only exon 4 which encoded the mature peptide was present [33]. 

Cathelicidins are expressed early on in development and throughout fish development [34], suggesting they play a role in immunity early on. In adults, cathelicidin expression is highly variable and is dependent on the cathelicidin gene, tissue and fish species examined. For example, cathelicidin 1 is not normally expressed in rainbow trout tissues but can be induced by pathogen challenge [27,31,32], whereas *S. trutta fario* cathelicidin 1 is highly expressed in the head kidney, trunk kidney and spleen, followed by the skin, gill and stomach of normal animals, but not detected in the brain or the testis [25]. However, cathelicidin 2 genes appear to be ubiquitously expressed in tissues such as the gill, kidney, intestine, skin and spleen [31,32,33]. In fish species such as Arctic charr with only one cathelicidin gene, cathelicidin shows constitutive expression with highest levels present in the skin, spleen and kidney, but also detected in the gill, liver, pyloric caeca, intestine, brain, heart and muscle [24]. Antibodies directed towards rainbow trout cathelicidins found cathelicidin peptides localized in the gut mucosa and around the sinusoids of the head kidney, associated with the lymphoid cells present in these areas [33]. Thus, cathelicidins in fish appear to be broadly distributed, however their location deviates with fish species and cathelicidin class.

Fish cathelicidins have broad-spectrum activity against Gram-negative bacteria, Gram-positive bacteria [27,28,29,31,33,35], and fungi [35]. A comprehensive list of antimicrobial activity, MICs and pathogen species can be found in [4]. Similar to the observations regarding transcript distribution, the antimicrobial activity of fish cathelicidins is highly variable depending on the fish species, pathogen, and particular cathelicidin. For example, while rainbow trout cathelicidins are active against *Y. ruckeri*, Atlantic salmon cathelicidins are not [31]. Initial studies in rainbow trout have demonstrated cathelicidins to bind LPS in a dose-dependent manner and exert their antimicrobial activity through membrane permeabilization [33]. Deletion studies in rainbow trout also showed the N-terminus of the mature cathelicidin peptides to be important for antimicrobial activity, although the extent of loss of activity in these deletion mutants was variable depending on the cathelicidin gene [33]. The activity of cathelicidins likely occurs as a result of the hypervariable nature of the mature cathelicidin peptide, suggesting their structural differences at the C-terminus are important in determining activity and selectivity against pathogens.

### 3.2. Regulation of Cathelicidins by Pathogens/Exogenous Factors

The expression of cathelicidin transcripts can be induced by pathogens and pathogen components in a time dependent manner and is species and gene copy dependent [36]. The promoter region of cathelicidin contains predicted sites for NF-IL-6, C/EBPβ, α-IRE, γ-IRE, NF-1 and NF-κB transcription factor binding sites [27,37], further supporting the role of cathelicidin as an immune responsive gene in fish. Indeed, cathelicidins transcript levels are induced *in vivo* in response to an array of Gram-negative and Gram-positive bacteria (Table 1, Table 2, Table 3 and Table 4) primarily in the spleen, kidney, gill, liver and intestine in a time dependent manner, usually within 24 h post challenge [24,27,31,32,38]. Treatment of rainbow trout cell lines with *Saprolegnia parasitica* revealed the differential regulation of cathelicidin 1 *versus* cathelicidin 2*;* cathelicidin 1 transcripts were upregulated in the rainbow trout cell lines RTL and RTS11 while cathelicidin 2 increased in RTG-2, RTGill, RTL and RTS11 rainbow trout cell lines [39]. Similar *in vitro* studies have demonstrated a time-, dose-, and cathelicidin class dependent increase in cathelicidin transcripts in Chinook salmon embryo cell lines (CHSE-214) in response to live bacteria, bacterial DNA and flagellin, occurring within 12 to 24 h post challenge [30,36]. Increasing amounts of bacteria led to a concentration-dependent increase in cathelicidin transcripts and this increase was time dependent, peaking at 24 h post challenge and decreasing sharply thereafter [36]. Furthermore, the Golgi apparatus, microtubules and PI3K signaling pathway appear to be important for inducing cathelicidin transcription in CHSE-214 fish cells in response to flagellin [36], suggesting a role for TLR5 and the downstream signaling pathway in the regulation of cathelicidin transcription. Cathelicidin transcript levels are also increased in response to pro-inflammatory cytokines such as IL-1β1 and IL-6 in rainbow trout macrophages and RTL cells [39,40]. Exogenous molecules can also influence cathelicidin transcript levels. For example, zymosan treatment of the rainbow trout intestinal epithelial RTgutGC cell line with zymosan *in vitro* or feeding rainbow trout a zymosan supplemented diet induced cathelicidin and IL-1β expression which were co-localized in the intestinal epithelial cells *in vivo* [41]. In addition, treatment of Atlantic salmon with 17β-estradiol or testosterone decreased the overall parasite load of the salmon louse ectoparasite and these fish were found to have higher mRNA levels of cathelicidin suggesting that cathelicidin may be an important factor in decreasing parasite load [42]. The modulation of cathelicidin in response to pathogens, pathogen components, inflammatory cytokines, and hormones suggest a role for cathelicidins in the immune response of fish towards pathogens.

### 3.3. Modulation of Immunity by Cathelicidins

Limited studies have been performed on teleost cathelicidins regarding their immunomodulatory role in fish. Both rainbow trout [33] and Atlantic salmon [31] cathelicidins induce the expression of IL-8 in fish peripheral blood leukocytes (PBLs) at early, but not later, time points following treatment of cells with cathelicidins [31]. However, Atlantic salmon cathelicidins did not induce IL-1 or IL-18 transcript levels, suggestive of selective induction of pro-inflammatory cytokines involved in chemotaxis [31]. Lastly, rainbow trout cathelicidin 2 treatment of rainbow trout RTgutGC cells was shown to induce IL-1β transcript levels [41]. Taken together, it appears that the regulation of pro-inflammatory transcripts by cathelicidins in fish occurs rapidly and is species, cathelicidin and cell type specific. Future studies on the immunomodulatory properties of teleost cathelicidins are needed in order to elucidate the role of cathelicidins in fish immunity. 

## 4. Piscidins 

### 4.1. Gene and Protein Structure, Expression and Function

Members of the piscidin family include piscidin, pleurocidin [43], moronecidin, misgurin, epinecidin [44], gaduscidin [45] and dicentracin and while they share an overall similar structure, they share little sequence homology amongst the mature peptides. Piscidin and piscidin family member genes have been identified in a number of fish species and include one piscidin family member in seabass (*Dicentrarchus labrax*) [46], icefish (*Chionodraco hamatus*) [47], rock bream (*Oplegnathus fasciatus*) [48], large yellow croaker (*Larimichthys crocea*) [49,50], striped trumpeter (*Latris lineata*) [51], mandarin fish (*Siniperca chuatsi*) [43], yellowtail flounder (*Pleuronectes ferruginea* Storer), American plaice (*Hippoglossoides platessoides* Fabricius), witch flounder (*Glyptocephalus cynoglossus* L.), Atlantic halibut (*Hippoglossus hippoglossus* L.) [52]; two in Atlantic cod (*Gadus morhua*) [45,53] along with a novel splice variant [54]; three in grouper (*Epinephelus coioides*) [44]; four in hybrid striped bass (*Morone chrysops* x *Morone saxatilis*) [55,56,57]; four genes and 3 pseudogenes in the winter flounder (*Pseudopleuronectes americanus*) [58,59]; and five in Nile tilapia (*Oreochromis niloticus*) [60]. The majority of the piscidin genes have a four-exon/three-intron genomic organization [43,55,61]. However, deviations from the common organization exist and include a three-exon/two-intron structure in one piscidin gene from Nile tilapia (*Oreochromis niloticus*) [60] and the yellow croaker (*Larimichthys crocea*) [49,50], as well as two epinecidin genes with a five-exon/four-intron structure in the grouper (*Epinephelus coioides*) [44]. 

Piscidins are produced as a 64–89 amino acid prepropeptide that undergoes proteolytic cleavage at the N-terminus to remove the signal peptide and at the C-terminus to cleave away the prodomain, releasing a 18–26 amino acid mature peptide that is ~2.5 kDa [43,55,56,60,61,62]. However, the piscidin 4 genes result in a 44 amino acid mature peptide that is ~5.5 kDa [56,57]. The mature piscidin peptides have an amphipathic α-helical conformation with the hydrophilic side being cationic and capable of interacting with pathogen membranes in a parallel fashion [48,62,63,64]. Collectively, piscidin family members seem to be undergoing positive selection [53,65], suggesting they may be undergoing diversification as a means to adapt to rapidly changing pathogens and would support the low sequence identity amongst the mature peptides of piscidin family members.

Differential tissue expression of piscidin genes within a species and also between fish species exists [58,59]. Piscidins are expressed early during fish development and generally increase in expression with fish development [54,58,59]. In adult tissues, piscidin transcripts and peptides are usually found at high levels in the skin, skin mucus, gill, intestine/gastrointestinal tract, head kidney, and spleen [43,45,47,49,50,54,57,58,59,66], while some piscidins are expressed more broadly and can also be detected in the liver, blood, gall bladder, pyloric caeca, stomach, rectum, muscle, heart, and brain [50,54,58,60]. Within these tissues, expression of piscidins have been detected in blood leukocytes, head kidney and spleen phagocytes, kidney hematopoietic cells [67], epithelial and basal cells of the dorsal skin, epithelial cells in the gill, multi-granular cells in the muscle, chondrocytes from cartilaginous tissues, epithelial columnar cells and multi-granular cells in the intestine, liver hepatocytes, and neuronal cells in the swim bladder [67]. However, piscidins are most commonly found in mast cells from the gills [46,47,51,68] at concentrations that are found to be inhibitory to fish pathogens *in vitro* [68,69], in mast cells and rodlet cells of the skin, gill and intestine [70], in eosinophilic granular cells [71], epithelial mucous cells [55,61,62], intestinal goblet cells [61,62], and in phagocytic granulocytes of the intestinal mucosa [72]. Furthermore, upon phagocytosis of bacteria by intestinal phagocytic granulocytes, the piscidin-containing granules fused with the bacteria-filled phagosome [72]. Together, these results support the involvement of piscidins in mucosal innate immunity [61], that piscidins are produced at sufficient concentrations *in vivo* at mucosal locations to have direct antimicrobial activity, and that they play an important inflammatory role in the elimination of pathogens. Furthermore, the lowered levels of piscidin in diseased and stressed fish [69] suggests the regulation of piscidin *in vivo* may be used as a marker for the health status of fish. 

Piscidin family members demonstrate antimicrobial or growth inhibition activity towards a wide array of Gram-negative and Gram-positive bacteria [48,52,54,55,56,58,60,62,73] with differing bacterial selectivity in a time-dependent fashion [62] and over a considerable temperature and salt range [55]. Piscidins also possess anti-viral activity under a broad range of physiological conditions and temperatures [74], anti-fungal activity [75,76], anti-parasite activity at physiological concentrations of piscidins found in fish species [49,54,77,78,79] and anti-tumor activity towards mammalian cells [80,81,82,83]. Piscidins can also act in synergy with other AMPs to enhance their activity [61,84,85]. Studies on the mechanism of action surrounding the anti-bacterial activity exhibited by piscidins show they interact with acidic phospholipids and form toroidal pores in the membrane [86,87,88]. While the extent of anti-fungal and anti-mammalian tumor activity varies with the piscidin gene expressed, the piscidin anti-fungi and anti-mammalian tumor activity appears to occur through membrane permeabilization and pore formation as well as the induction of reactive oxygen species and apoptotic pathways [75,76,80,81,82,83,89]. The *in vitro* antimicrobial studies performed support piscidins as direct defense molecules against a wide array of pathogens *in vivo* and their key role in the fish immune system.

### 4.2. Regulation of Piscidins by Pathogens/Exogenous Factors

The upstream promoter regions of members of the piscidin family contain putative binding sites for NF-IL-6, alpha-IRE, gamma-IRE [58,61], AP-1 [58], C/EBPβ [55] and hepatocyte nuclear factor (HNF-1) [44] transcription factors, with HNF-1 shown to be required for the transcriptional activation of epinecidin in response to LPS [44]. Although the receptors, intracellular signaling pathway and transcription factors involved in the regulation of piscidin family members in response to pathogens have yet to be explicitly identified and characterized, it appears that LPS [43], Gram-negative and Gram-positive bacteria [45,47,48,54,60,66,90], poly I:C [47,66], viruses [47,48] and parasites [46,51,91,92,93,94] enhance the expression of piscidin family members in fish spleen, kidney, skin, and intestinal tissues as well as in mast cells (Table 1, Table 2, Table 3 and Table 4 and references therein). However, it is important to note that the regulation of piscidin transcript and protein levels is pathogen, fish, tissue and piscidin dependent, thus requiring detailed studies of each fish species, pathogen, tissue, and piscidin family member. Despite the general induction of piscidin family members in fish challenged with pathogens, some systems exist in which piscidin family members are not induced in response to pathogens such as the case with moronecidin transcripts which remained unchanged in hybrid sea bass challenged with *S. iniae* [55]. That fish piscidin family members are largely inducible in response to a broad array of pathogen or pathogen components provides support for their essential role in the immune response for defense against pathogens. 

### 4.3. Modulation of Immunity by Piscidins

Members of the piscidin family possess immunomodulatory activity. *In vitro* studies have shown treatment of the rainbow trout RTS11 macrophage cell line with pleurocidin to induce the expression of IL-1β and COX1 transcripts in a dose and time dependent manner, but did not impact Mx, antigen presentation or JAK/STAT gene expressions [73]. However, unlike that of other AMPs, treatment of RTS11 fish cells with LPS and pleurocidin did not affect the LPS inflammatory induced response [73]. Meanwhile, studies in the cured barramundi brain (cBB) fish cell line showed grouper epinecidin-1 to induce Mx transcripts [84]. Thus, it appears that induced gene expression is dependent on the piscidin family member and the cell line employed. 

Collectively, *in vivo* studies have shown piscidins to aid in inhibiting pathogen replication, lowering pathogen load and increasing host survival in a number of fish species when challenged with bacteria such as *Vibrio vulnificus* [95,96,97,98] or viruses such as nervous necrosis virus (NNV) [84,99,100]. While these studies share the overall finding of piscidin protection of fish against pathogens, the mechanism of how this is achieved appears to vary depending on the piscidin gene, route of piscidin administration, fish species and pathogen used for challenge. For example, in grouper (*Epinephelus coioides*) Mx2 and Mx3 transcript levels were down regulated in response to piscidin [84]. Meanwhile, transgenic zebrafish expressing epinecidin-1 showed induced MyD88 expression [98], which differed from zebrafish injected with epinecidin-1 that induced IL-10, IL-15, IL-17c, and MAPK gene expressions but failed to induce any changes in MyD88, TLRs, IFNγ, IL-1β, IL-12, IL-4/IL-13A, leptin, NF-κB, SP1, TNF-α or REL-A [95]. Interestingly, in a study examining the differential expression of proteins in zebrafish injected with epinecidin-1, researchers failed to identify any differences in immune proteins and instead found changes in the levels of proteins involved in cell assembly, organization, function, and maintenance [101]. Finally, when fish were fed *E. coli* expressing epinecidin-1, enhanced expressions of TNF, TLR4, IL-1β, nitric oxide synthase 2, and NF-κB were observed [96]. However, in this case it is difficult to discern whether the gene expressions observed were solely the result of epinecidin-1 or whether the *E. coli* in conjunction with epinecidin-1 were responsible for the transcriptional activation of key players in the pro-inflammatory response. Despite the studies performed on fish piscidins and their immunomodulatory role in fish, the immunomodulatory roles of fish piscidins remains somewhat clouded and further studies are needed to elucidate the mechanism of action in which piscidins appear to confer resistance towards a number of pathogens *in vivo*. 

## 5. Hepcidins 

### 5.1. Gene and Protein Structure, Expression and Function

Hepcidin was originally identified in humans [102,103], but since then has been identified in a number of vertebrates. In mammals, hepcidin is expressed primarily in the liver and plays an important role in maintaining iron homeostasis as well as possessing direct antimicrobial activity towards pathogens *in vitro*, reviewed by [104]. In fish, the hepcidin gene has a conserved three-exon/two- intron structure [9,13,14,22,23,24,25,26,27]. Although a single hepcidin gene exists in humans, whole genome duplications in fish have led to some fish species having multiple copies of hepcidin. The number of genomic copies of hepcidin varies in each species of fish; one copy has been found in the large yellow croaker (*Pseudosciaena crocea*) [105], channel catfish (*Ictalurus punctatus*) [106,107], hybrid striped bass [106,108]; two copies in zebrafish (*Danio rerio*) [109], medaka (*Oryzias melastigmus*) [110], turbot (*Scophthalmus maximus*) [111], Atlantic salmon (*Salmo salar*, L.) [112], rainbow trout (*Oncorhynchus mykiss*) [113], olive flounder (*Paralichthys olivaceus*) [114], black rockfish (*Sebastes schlegelii*) [115], barramundi (*Lates calcarifer*) [116]; three copies in tilapia (*Oreochromis mossambicus*) [117], orange-spotted grouper (*Epinephelus coioides*) [118,119]; four copies in rockbream (*Oplegnathus fasciatus*) [120], six-to-eight copies in seabass (*Dicentrarchus labrax*) [121]; seven copies in the black porgy (*Acanthopagrus schlegelii* B) [122] and nine copies that fall into five categories in the winter flounder (*Pseudopleuronectes americanus*) [112]. Hepcidins have been identified in over 37 species of fish and a comprehensive table outlining the number of hepcidins genes in each species is reviewed in [4]. 

Fish hepcidins are encoded as prepropeptides having three domains; a 22–24 amino acid leader domain, a 40–47 amino acid proregion domain, followed by a 20–26 amino acid mature peptide domain that contains the 6-8 cysteine residues that are important in the formation of the 3-4 disulphide bonds [105,117,123,124,125,126], yielding a 2-3 kDa mature peptide. However, it should be noted that some fish hepcidins only possess four cysteine residues in their mature peptides [105], such as the red-spotted grouper (*Epinephelus akaara*) [127], the orange-spotted grouper (*Epinephelus coioides*) [118,119] and rainbow trout (*Oncorhynchus mykiss*) [113]. Sequences of the mature hepcidin peptides from fish to mammals show high conservation of the 8 cysteine residues found within the mature peptide, with one of the four disulphide bridges being a vicinal disulphide bridge, are rich in beta sheets and cationic with a tendency to primarily form amphipathic molecules [105,113,114,117,121,125]. Conservation of the cysteine residues required for disulphide bridges points to their functional importance in conferring antimicrobial activity towards pathogens and their amphipathic nature is likely important for the interaction with and insertion of hepcidins into microbial membranes or interaction with intracellular components. 

Expression of hepcidins occurs early during development and in some fish species appears to increase in expression with development [106,124,127]. In adult fish, hepcidin is primarily expressed in the liver, as is the case in the red-spotted grouper (*Epinephelus akaara*) [127]. However, depending on the fish species and the isoform, hepcidin can also be found expressed in the skin, gill, head kidney, trunk kidney, intestine, muscle, ovary, gonad, heart, spleen, brain, stomach, blood and peritoneal leukocytes [106,107,108,121,123,128]. Furthermore, studies in gilthead seabream demonstrated that hepcidin is most highly expressed in acidophilic granulocytes compared to monocytes/macrophages and lymphocytes [128]. 

In those fish species that have multiple hepcidin isoforms, expression is tissue specific, displaying differing patterns of expression between fish for similar isoforms, tissue specific for multiple isoforms within the same fish species and their regulation in response to pathogen challenge is also isoform specific [116,119,120,129,130,131]. It is proposed that the multiple copies of hepcidin have arisen due to the whole genome duplications that have occurred in fish and that the dual role of mammalian hepcidin has been sub-functionalized between the hepcidin copies observed in fish, *i.e.* one hepcidin has iron regulatory roles while the second hepcidin copy has retained the antimicrobial role [114,132,133]. This is supported by the inducible expression of one hepcidin copy while the other lacks inducible expression in response to pathogen challenge and rather appears to be iron responsive [114,117,119,133,134]. In those fish with only a single hepcidin gene, the dual function in iron homeostasis and as an AMP appears to be conserved [135]. However, for fish species that have greater than two hepcidin isoforms, the sub-functionalization or neo-functionalization of these isoforms have yet to be clearly elucidated. It appears that positive selection is occurring in the hepcidins of fish [136,137]. 

Collectively, the hepcidins of fish possess antimicrobial activity towards a broad range of pathogens *in vitro*, including Gram-negative bacteria, Gram-positive bacteria, viruses, and parasites and demonstrate varying minimal inhibitory concentrations depending on the particular pathogen, the fish species the hepcidin is derived from, and the hepcidin isoform itself. The antimicrobial activity of recombinant AMPs and synthesized AMPs from a number of fish species, in a dose-dependent manner, has been demonstrated [111,126,138]. A comprehensive list of the MICs associated with each fish hepcidin and the pathogen they demonstrate antimicrobial activity against can be found in [4]. 

Studies examining the structure-function relationship of fish hepcidins have shown a number of key features to be necessary for antimicrobial function. In zebrafish, the cysteine residues in a recombinant hepcidin-2 peptide were found to be essential for the binding of recombinant zebrafish hepcidin-2 to LPS, LTA and PGN and direct antimicrobial effects observed, suggesting that zebrafish hepcidin-2 acts as a pattern recognition molecule [126]. Studies on trout hepcidin revealed an amine terminal Cu^2+^ and Ni^2+^ binding (ATCUN) motif that is important for metal binding and was essential for DNA hydrolysis [139]. However, the truncation of this ATCUN motif in trout hepcidin did not result in loss of antimicrobial activity towards *E. coli* and *P. salmonis* [139]*.* Membrane permeabilization studies and confocal microscopy suggested that the mechanism of bacterial growth inhibition observed in response to trout hepcidin was not through bacterial membrane permeabilization or destruction, but rather appeared to be through the interaction with the bacterial DNA, regardless of the presence or absence of the ATCUN motif [139]. However, another study on turbot hepcidins found turbot hepcidin to be capable of binding to *E. tarda* and this interaction caused the bacteria to look transparent as a result of alteration of the bacterial surface, suggesting that turbot hepcidins were disrupting the outer membrane of the bacteria [111]. Furthermore, pre-treatement of *E. tarda* with turbot hepcidins prior to infection of flounder FG fish cells greatly reduced the number of viable intracellular bacteria present in the cell cultures [111]. Collectively, these studies demonstrate key structural properties of fish hepcidins and the multiple mechanisms in which they can exert their microbial action. 

A few studies have demonstrated the antimicrobial activity of fish hepcidin *in vivo.* Prior *in vivo* administration of synthetic tubort hepcidins greatly reduced the *E. tarda* bacterial load or megalocytivirus load recovered from the kidney, spleen and liver of infected fish compared to that of non-treated infected control fish [111]. Studies have also employed the use of transgenic zebrafish for the over expression of tilapia hepcidin 1-5 (TH1-5) in live animals. Overexpression of TH1-5 in transgenic zebrafish conferred resistance to challenge with *V. vulnificus* or *S. agalactiae* compared to wildtype zebrafish [140,141]. Lastly, *in vitro* studies in hybrid striped bass demonstrate synergy between antimicrobial peptides [142], suggesting that the current manner in which *in vitro* and *in vivo* studies are performed (*i.e.,* measuring MICs for single AMPs at a time) may not be reflective of the actual physiological conditions found within an organism that often expresses more than a single AMP. Although these studies do demonstrate the antimicrobial capacity of fish AMPs *in vivo*, the levels at which the AMPs were artificially simulated may be outside of the physiological range of AMP expression in wildtype fish, or may require “partner” AMPs in order to attain optimal activity. Further studies are required in order to ascertain whether AMPs have a direct antimicrobial action on fish pathogens under physiological conditions. 

### 5.2. Regulation of Hepcidin by Pathogens/Exogenous Factors

In accord with the role of hepcidin in iron homeostasis in mammals, fish hepcidin is upregulated in response to iron or anemia and can be modulated by transferrin [104,121,135,143,144], suggesting that hepcidin isoforms also participates in iron homeostasis in fish. Furthermore, hepcidin expression in fish can be rapidly upregulated in response to IL-6 and bone morphogenic protein (BMP) [40,145,146,147], bacterial components such as LPS, Gram-negative bacteria, Gram-positive bacteria, viruses or viral mimics and parasites (see Table 1, Table 2, Table 3 and Table 4 and references therein), suggesting that hepcidin is an acute phase protein and involved in an inflammatory response akin to that of hepcidin in mammals [104,148,149]. The tissues in which hepcidin expression can be induced in response to pathogen challenge are broad, often depending on the fish species and hepcidin isoform studied. For example, bass hepcidin is upregulated to the greatest extent in the liver and to a lesser extent in the skin, gill, intestine, spleen, kidney and blood in *Streptococcus iniae* challenged fish [108]. However in some fish species such as catfish, hepcidin levels in the liver did not increase upon bacterial challenge, yet increased instead in kidney and spleen tissues [106]. The transcriptional activation of fish hepcidins in response to iron levels, IL-6, and pathogens is in accord with the presence of putative binding sites for the HNF, STAT, NF-κB, and C/EBP transcription factors found in the fish hepcidin promoter regions [106,108,109,120], as IL-6 signals primarily though the JAK/STAT pathway and detection of pathogens and pathogen components can occur via TLRs and downstream NF-κB activation. It is interesting to note that fish hepcidins can also be modulated in response to environmental contaminants [150,151] and high temperatures [152], implicating environment impacts on the potential health of fish. Together, these studies support the role of hepcidin as an APP required for the sequestering of iron during an immune response to limit pathogen growth and as an AMP with direct antimicrobial activity towards pathogens, both of which act together to limit the spread of infection.

### 5.3. Modulation of Immunity by Hepcidin

Fish hepcidins, in addition to having iron-regulating and antimicrobial properties, have demonstrated immune-modulating properties, albeit limited studies have been performed demonstrating this attribute. Simple *in vitro* studies in which Chinook salmon embryo (CHSE-214) cells were treated with tilapia hepcidin 1-5 (TH1-5) lead to an immediate (2 h post treatment) induction of annexin and metallothionein mRNA levels [153], but returned to basal levels 16 h post treatment. *In vivo* studies in which transgeneic zebrafish overexpressing TH1-5 were challenged with *V. vulnificus* and *S. agalactiae* exhibited enhanced resistance to infection with these pathogens [140,141]. Examination of immune gene regulation between the transgenic and wildtype zebrafish revealed that transgenic TH1-5 zebrafish exhibited higher mRNA levels of IL-10, IL-21, IL-22, lysozyme, NF-κB, TNF-α and TLR-1 compared to that of wildtype zebrafish. These data suggest that TH1-5 acts to upregulate these immune molecules in fish, leading to immunoregulation in the host that confers resistance to *V. vulnificus* and *S. agalactiae in vivo*. [140,141]. In some cases, fish hepcidins are used to treat mammalian cells in attempts to elucidate their immunomodulatory properties and the pathways through which they work. Treatment of mammalian RAW264.7 macrophage cells with tilapia hepcidin 2-3 (TH2-3) induced a elongated shape in mammalian RAW264.7 cells, found to be triggered through PKC (inhibits PKCµ-744 phosphorylation but promotes PKCµ-916 phosphorylation) and MEK pathways and resulted in a decrease in the mRNA levels of markers associated with dendritic cells [153,154]. However, the action of TH2-3 also appeared to abrogate the dendritic like shape induced by LPS in the mammalian RAW264.7 cells and was found to suppress TNF-α, IL-1α, IL-1β, IL-6, and cyclooxygenase-2 (COX-2) expression in the presence of LPS [153,154]. Despite the latter study using a fish hepcidin in a mammalian model system, these data seem to suggest that fish hepcidin may act as an immunostimulatory peptide in the absence of pathogen components, but may act to immunomodulate and dampen a potentially exaggerated an harmful pro-inflammatory response in the presence of bacterial components. Thus, it appears that hepcidin may also act as an important HDP to regulate inflammation. 

## 6. Histone-Derived Peptides 

Histone-derived peptides are >10 kDa peptide fragments generated from the proteolytic cleavage of histones and have been identified in fish. Fish histone-derived peptides originate from both the N-terminus and C-terminus of H1 [85,155,156,157] and H2A [157,158,159] histones and are found in the skin and skin mucus [85,155,158,160]. Limited studies on fish histone-derived peptides have revealed that they can be produced in response to epidermal damage, LPS or certain Gram-negative bacteria (Table 1, Table 2, Table 3 and Table 4) [159] and have antimicrobial activity against a broad spectrum of Gram-positive and Gram-negative bacteria [85,155,156,158], parasites [160] and fungi [157], with a list of the MICs for histone-derived peptides towards Gram-negative and Gram-positive bacteria in [4]. In one case, a histone-derived peptide without direct antimicrobial activity on its own was capable of potentiating the activity of other AMPs [85], supporting the idea that some AMPs may require a partner AMP in order to achieve maximal and potent antimicrobial activity. Few studies have examined the mechanisms of antimicrobial activity exerted by histone-derived peptides. However, histone-derived peptides in their active form are more structured, rigid and condensed than their inactive forms [156] and they bind to anionic membranes [85]. While some histone-derived peptides bind and permeabilize the membrane [157,161], others do not, suggesting they require a binding partner to enter the cell [85]. In comparison to other fish AMPs such as hepcidin, relatively little is known about the histone-derived peptides in fish in terms of their distribution across species of fish, conservation of cleavage sites, the signal that triggers the cleavage of the histone-derived peptides from histones, their antimicrobial mechanism of action on pathogens and whether these histone-derived peptides can act as host-defense peptides and modulate immune responses.

**Table 1:** Gram-negative pathogens that modulate the transcript levels of antimicrobial peptides in fish tissues.

Pathogen	β-Defensin	Cathelicidin	Piscidin	Hepcidin	Histone-Derived
*Aeromonas hydrophilia*	[ 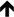 ] eye, gill, skin, spleen (*Paramisgurnus dabryanus*) [10]				
*Aeromonas salmonicida*		[ 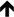 ] spleen, gill, head kidney (*Oncorhynchus mykiss*) [27,32] [ 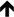 ] CHSE-214 cells (*Onchorhynchus tshawytscha*) [30] [ 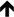 ] spleen, head kidney, gill, liver (*Gadus morhua*) [24,34,38,162] [ 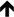 ] liver, pyloric caeca, intestine, gills (*Gadus morhua* [24] [ 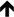 ] skin, liver, pyloric caeca, intestine, gills, heart, muscle (*Salvelinus alpinus*) [24]	[ 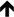 ] head kidney, spleen (*Gadus morhua*) [45]	[ 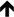 ] liver, spleen, intestine, kidney, muscle, stomach esophagus (*Salmo salar, L.*) [112,163] [ 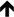 ] intestine, head kidney (*Oncorhynchus mykiss*) [113,164] [ 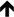 ] liver (hybrid striped bass) [142] [ 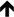 ] internal organs (*Danio rerio*) [148] [ 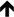 ] head kidney, spleen (*Gadus morhua*) [38]	
*Escherichia coli*		[ 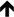 ] CHSE-214 cells (*Onchorhynchus tshawytscha*) [30]		[ 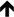 ] spleen, head kidney, gill, intestine, liver (*Chrysophrys major*) [123] [ 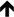 ] RTS11 macrophage cell line (*Oncorhynchus mykiss*) [165] [ 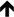 ] liver, kidney, spleen, intestine (*Oplegnathus fasciatus*) [120]	
*Edwardsiella ictaluri*				[ 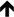 ] spleen, kidney (*Ictalurus punctatus*) [106] [ 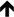 ] spleen, liver, intestine (*Ictalurus punctatus*) [107,134,135]	
*Edwardsiella tarda*	[ 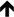 ]head kidney (*Paralichthys olivaceus*) [17] [ 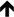 ]intestine (*Danio rerio*) [9]		[ 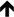 ]spleen, [ 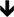 ] kidney (*Oplegnathus fasciatus*) [48]	[ 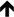 ] PBLs (*Paralichthys olivaceus*) [166] [ 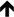 ] liver, kidney, spleen, intestine (*Oplegnathus fasciatus*) [120]	
LPS	[ 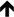 ] eye (*Oryzias latipes*) [11] [ 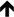 ] spleen, liver (*Epinephelus coioides*) [18]	[ 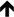 ] head kidney leukocytes, head kidney, gut (*Oncorhynchus mykiss*) [27,33] [ 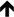 ] CHSE-214 (*Onchorhynchus tshawytscha*) [30]	[ 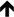 ] brain, heart, gill, kidney, pronephros, skin, spleen, intestine (*Siniperca chuatsi*) [43] [ 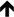 ] head kidney leukocytes (*Chionodraco hamatus*) [47] [ 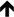 ] head kidney, intestine, skin (*Epinephelus coioides*) [66]	[ 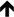 ] liver, spleen, head kidney, gill (*Paralichthys olivaceus*) [114,133] [ 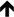 ] liver, intestine, kidney, (*Oreochromis mossambicus*) [117] [ 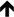 ] head kidney leukocytes (*Sparus aurata)*[128] [ 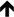 ] spleen, heart, stomach, kidney, liver (*Pseudosciaena crocea)*[105] [ 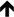 ] liver, kidney (*Lates calcarifer*) [116] [ 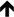 ] liver (*Oncorhynchus mykiss*) [167] [ 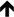 ] liver, stomach, heart, intestine, gill, muscle (*Amatitlania nigrofasciata*) [168]	[ 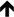 ] mucus (*Oncorhynchus kisutch*) [85]
*Photobacterim damselae*				[ 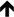 ] liver (*Dicentrarchus labrax*) [121]	
*Pseudomonas aeruginosa*		[ 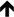 ] CHSE-214 cells (*Onchorhynchus tshawytscha*) [36]			
*Vibrio anguillarum*	[ 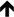 ] spleen, gills, skin, foregut, hindgut (*Cyprinus carpio*) [14] [ 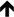 ] head kidney (*Gadus morhua*) [8]	[ 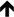 ] CHSE-214 cells (*Onchorhynchus tshawytscha*) [30,36] [ 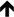 ] liver, spleen, head kidney, gill, intestine, muscle (*Plecoglossus altivelis*) [28]	[ 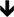 ] skin, gills, [ 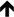 ] head kidney (*Dicentrarchus labrax*) [90]	[ 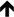 ] liver (*O. mykiss*) [149] [ 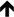 ] liver, spleen (*Scophthalmus maximus*) [124] [ 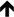 ] liver, spleen, peritoneal leukocytes (*Sparus aurata)*[128] [ 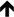 ] liver, head kidney, peritoneum (*Gadus morhua* L.*)*[125] [ 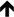 ] liver, kidney, spleen, intestine, (*Oplegnathus fasciatus*) [120] [ 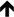 ] liver, kidney, spleen, intestine, skin (*Cyprinus carpio* L.) [131,169]	[ 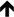 ] gills (*Dicentrarchus labrax*) [90]
*Yersinia ruckeri*	[ 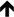 ] intestine, gill (*Oncorhynchus mykiss*[16]	[ 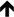 ] CHSE-214 cells (*Onchorhynchus tshawytscha*) [ 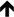 ] gill, spleen (*Salmo salar*) [30,31] [ 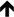 ] fry (*Oncorhynchus mykiss*) [170]			

**Table 2:** Gram-positive pathogens that modulate the transcript levels of antimicrobial peptides in fish tissues.

Pathogen	β-Defensin	Cathelicidin	Piscidin	Hepcidin	Histone-Derived
*Lactococcus garvieae*				[ 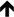 ] liver, kidney, spleen, intestine (*Oplegnathus fasciatus*) [120]	
*Staphylococcus aureus*				[ 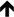 ] internal organs (*Danio rerio*) [148]	
*Streptococcus agalactiae*	[ 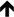 ] skin, muscle, kidney, gill (*Oreochromis niloticus*) [20]				
*Streptococcus iniae*			[ 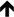 ]kidney, spleen, gill (*Oplegnathus fasciatus*) [48]	[ 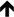 ] liver, skin, gill, intestine, spleen, kidney, blood (*Morone saxatilis*) [108], [ 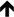 ] abdominal organs (*Danio rerio*) [109] [ 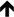 ] liver (hybrid striped bass) [142] [ 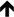 ] kidney, gill, spleen, brain, liver, skin (*Sebastes schlegelii*) [115] [ 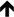 ] liver, kidney, spleen, intestine (*Oplegnathus fasciatus*) [120]	

**Table 3:** Viruses that modulate the transcript levels of antimicrobial peptides in fish tissues.

Pathogen	β-Defensin	Cathelicidin	Piscidin	Hepcidin	Histone-Derived
Cyprinid herpesvirus-3	[ 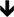 ] skin (*Cyprinus carpio*) [22]				
Infectious hematopoietic necrosis virus				[ 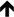 ] RTS11 macrophage cell line (*Oncorhynchus mykiss*) [165]	
Lymphocystis iridovirus			[ 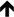 ] acidophilic granulocytes (*Sparus aurata*) [93]		
Nodavirus (VNNV)				[ 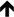 ] gonad, brain (*Dicentrarchus labrax*) [171]	
Poly I:C	[ 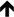 ] head kidney leuckocytes (*Oncorhynchus mykiss*) [16] [ 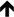 ] spleen, liver (*Epinephelus coioides*) [18]	[ 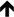 ] head kidney leukocytes (*Oncorhynchus mykiss*) [27]	[ 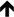 ] head kidney leukocytes (*Chionodraco hamatus*) [47] [ 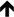 ] head kidney, intestine, skin (*Epinephelus coioides*) [66]	[ 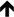 ] spleen (*Pseudosciaena crocea*) [121] [ 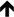 ] undisclosed tissue, (*Oreochromis mossambicus*) [117] [ 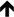 ] RTS11 macrophage cell line, spleen (*Oncorhynchus mykiss*) [165] [ 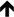 ] head kidney leukocytes (*Sparus aurata)*[128]	
Red seabream iridovirus (RSIV)			[ 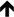 ]spleen, kidney (*Oplegnathus fasciatus*) [48]		
Singapore grouper iridovirus (SGIV)	[ 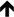 ] spleen, liver (*Epinephelus coioides*) [18]				
Viral hemorrhagic septicemia virus				[ 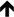 ] liver, head kidney, spleen (*Sparus aurata)*[128]	

**Table 4:** Parasites that modulate the transcript levels of antimicrobial peptides in fish tissues.

Pathogen	β-Defensin	Cathelicidin	Piscidin	Hepcidin	Histone-Derived
*Acanthocephalus lucii*			[ 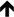 ] intestine mast cells (*Perca fluviatilis*)[92]		
*Chondracanthus goldsmidi*			[ 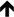 ] gills (*Latris lineata*) [51]		
*Cryptocaryon irritans*			[ 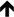 ] gills, skin, spleen, head kidney, liver, intestine (*Pseudosciaena crocea*) [49]	[ 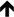 ] liver, kidney (*Lates calcarifer)*[172]	
*Ergasilus sp.*			[ 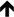 ] gill mast cells (*Sparus aurata*) [94]		
*Ichthyobodo necator*		[ 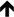 ] skin (*Oncorhynchus mykiss*) [173]			
*Ichthyophthirius multifiliis*		[ 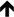 ] larvae (*Oncorhynchus mykiss*) [118,174]		[ 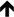 ] larvae (*Oncorhynchus mykiss*) [118]	

## 7. Conclusions 

The past 20 years of research on teleost AMPs have identified over 90 peptides from five classes of AMPs and reported on their genomic organization, copy number, conserved motifs and structures, transcript distribution over fish development and within tissues, antimicrobial activity towards an array of fish and human pathogens, and their expression in response to pathogens. Studies on teleost AMPs have only just begun to investigate the potential synergy between AMPs, their transcriptional regulation in response to host pro-inflammatory molecules, the immunomodulatory activities AMPs possess in the absence or presence of infection and the receptors, intracellular signaling molecules and transcription factors that regulate them. Of particular interest is the receptor(s) to which AMPs bind in fish to elicit an immunostimulatory response in the absence of pathogens and how this pathway converges with pathogen-activated pathways to then immunomodulate and dampen pro-inflammatory pathways in the presence of pathogens. It is intriguing to ponder if the binding of AMPs to pathogens at sub-MIC levels may act to opsonize the pathogen and enhance immune receptor signaling leading to immune activation and destruction of the AMP-bound pathogen in fish, particularly in light of their chemoattractive activity. It may be possible that there is a dose-dependency to the type of response elicited from teleost immune cells in response to AMPs. With over 28,000 species of teleosts, the large diversity of teleost AMPs, each with their own unique species-specific tissue/cell distribution, antimicrobial activity and immunomodulatory activity, in conjunction with the positive selection occurring on AMPs speaks to their adaptation to the fish-specific pathogens they defend against. In light of the expansive number of teleost species, it is likely that many fish AMPs will be identified in future studies. 

The immunomodulatory activity of teleost AMPs has broad application to fish health, particularly in aquaculture settings where high density rearing that are permissive for rapid pathogen transmission and stress-induced immunosuppression can occur. The addition of compounds, such as TLR ligands, to fish feed that induce mucosal AMP expression may provide fish with a means of protection against mucosal fish pathogens, either as a preventative measure or during active infection. However, further studies are required in order to optimize levels of AMP-inducers in fish feed to prevent disregulation in AMP expression in fish, perhaps leading to a prolonged inflammatory response and tissue destruction in fish if applied in too high of a dose or for too long. Furthermore, monitoring the expression of AMPs has the potential to be an indicator of fish health, allowing for appropriate and timely strategies to be employed to maintain fish health [175].

Teleost AMPs may also have applications in the medical field. Antibiotic resistant bacteria are increasing at an alarming rate, necessitating the development of other antimicrobial agents for the therapeutic treatment of patients. Of particular consideration is the use of AMPs as a replacement strategy for the failing antibiotics due to the demonstrated antimicrobial activity of teleost AMPs both *in vitro* and *in vivo*, broad specificity and ability to modulate the immune response in mammals [176,177,178]. However, while this is an emerging area, much research is still needed in order to translate the use of AMPs as antibiotic replacements into the medical field. 

## References

[1] Zasloff M. (2002). Antimicrobial peptides of multicellular organisms. Nature.

[2] Gilmore T.D., Wolenski F.S. (2012). NF-κB: Where did it come from and why?. Immunol. Rev..

[3] Wang G., Li X., Wang Z. (2009). APD2: The updated antimicrobial peptide database and its application in peptide design. Nucleic Acids Res..

[4] Masso-Silva J.A., Diamond G. (2014). Antimicrobial peptides from fish. Pharmaceuticals.

[5] Zou J., Mercier C., Koussounadis A., Secombes C. (2007). Discovery of multiple beta-defensin like homologues in teleost fish. Mol. Immunol..

[6] Cuesta A., Meseguer J., Esteban M.A. (2011). Molecular and functional characterization of the gilthead seabream beta-defensin demonstrate its chemotactic and antimicrobial activity. Mol. Immunol..

[7] Jin J.Y., Zhou L., Wang Y., Li Z., Zhao J.G., Zhang Q.Y., Gui J.F. (2010). Antibacterial and antiviral roles of a fish beta-defensin expressed both in pituitary and testis. PLoS ONE.

[8] Ruangsri J., Kitani Y., Kiron V., Lokesh J., Brinchmann M.F., Karlsen B.O., Fernandes J.M. (2013). A novel beta-defensin antimicrobial peptide in Atlantic cod with stimulatory effect on phagocytic activity. PLoS ONE.

[9] Liu X., Chang X., Wu H., Xiao J., Gao Y., Zhang Y. (2014). Role of intestinal inflammation in predisposition of *Edwardsiella tarda* infection in zebrafish (*Danio rerio*). Fish Shellfish Immunol..

[10] Chen Y., Zhao H., Zhang X., Luo H., Xue X., Li Z., Yao B. (2013). Identification, expression and bioactivity of Paramisgurnus dabryanus β-defensin that might be involved in immune defense against bacterial infection. Fish Shellfish Immunol..

[11] Zhao J.G., Zhou L., Jin J.Y., Zhao Z., Lan J., Zhang Y.B., Zhang Q.Y., Gui J.F. (2009). Antimicrobial activity-specific to Gram-negative bacteria and immune modulation-mediated NF-kappaB and Sp1 of a medaka beta-defensin. Dev. Comp. Immunol..

[12] Liang T., Wang D.D., Zhang G.R., Wei K.J., Wang W.M., Zou G.W. (2013). Molecular cloning and expression analysis of two β-defensin genes in the blunt snout bream (*Megalobrama amblycephala*). Comp. Biochem. Physiol. B Biochem. Mol. Biol..

[13] Marel M., Adamek M., Gonzalez S.F., Frost P., Rombout J.H., Wiegertjes G.F., Savelkoul H.F., Steinhagen D. (2012). Molecular cloning and expression of two beta-defensin and two mucin genes in common carp (*Cyprinus carpio* L.) and their up-regulation after beta-glucan feeding. Fish Shellfish Immunol..

[14] Li H., Guo H., Shan S., Qi C., An L., Yang G. (2014). Characterization and expression pattern of a novel β-defensin in common carp (*Cyprinus carpio* L.): Implications for its role in mucosal immunity. Biosci. Biotechnol. Biochem..

[15] Falco A., Chico V., Marroqui L., Perez L., Coll J.M., Estepa A. (2008). Expression and antiviral activity of a beta-defensin-like peptide identified in the rainbow trout (*Oncorhynchus mykiss*) EST sequences. Mol. Immunol..

[16] Casadei E., Wang T., Zou J., Gonzalez Vecino J.L., Wadsworth S., Secombes C.J. (2009). Characterization of three novel beta-defensin antimicrobial peptides in rainbow trout (*Oncorhynchus mykiss*). Mol. Immunol..

[17] Nam B.H., Moon J.Y., Kim Y.O., Kong H.J., Kim W.J., Lee S.J., Kim K.K. (2010). Multiple beta-defensin isoforms identified in early developmental stages of the teleost Paralichthys olivaceus. Fish Shellfish Immunol..

[18] Guo M., Wei J., Huang X., Huang Y., Qin Q. (2012). Antiviral effects of beta-defensin derived from orange-spotted grouper (*Epinephelus coioides*). Fish Shellfish Immunol..

[19] Wang G., Li J., Zou P., Xie H., Huang B., Nie P., Chang M. (2012). Expression pattern, promoter activity and bactericidal property of beta-defensin from the mandarin fish *Siniperca chuatsi*. Fish Shellfish Immunol..

[20] Dong J.J., Wu F., Ye X., Sun C.F., Tian Y.Y., Lu M.X., Zhang R., Chen Z.H. (2015). Beta-defensin in Nile tilapia (*Oreochromis niloticus*): Sequence, tissue expression, and anti-bacterial activity of synthetic peptides. Gene.

[21] Oehlers S.H., Flores M.V., Chen T., Hall C.J., Crosier K.E., Crosier P.S. (2011). Topographical distribution of antimicrobial genes in the zebrafish intestine. Dev. Comp. Immunol..

[22] Adamek M., Syakuri H., Harris S., Rakus K.L., Brogden G., Matras M., Irnazarow I., Steinhagen D. (2013). Cyprinid herpesvirus 3 infection disrupts the skin barrier of common carp (*Cyprinus carpio* L.). Vet. Microbiol..

[23] Cerezuela R., Guardiola F.A., Meseguer J., Esteban M.A. (2012). Enrichment of gilthead seabream (*Sparus aurata* L.) diet with microalgae: Effects on the immune system. Fish Physiol. Biochem..

[24] Maier V.H., Dorn K.V., Gudmundsdottir B.K., Gudmundsson G.H. (2008). Characterisation of cathelicidin gene family members in divergent fish species. Mol. Immunol..

[25] Scocchi M., Pallavicini A., Salgaro R., Bociek K., Gennaro R. (2009). The salmonid cathelicidins: A gene family with highly varied C-terminal antimicrobial domains. Comp. Biochem. Physiol. B Biochem. Mol. Biol..

[26] Garcia-Valtanen P., Martinez-Lopez A., Ortega-Villaizan M., Perez L., Coll J.M., Estepa A. (2014). In addition to its antiviral and immunomodulatory properties, the zebrafish beta-defensin 2 (zfBD2) is a potent viral DNA vaccine molecular adjuvant. Antiviral Res..

[27] Chang C.I., Pleguezuelos O., Zhang Y.A., Zou J., Secombes C.J. (2005). Identification of a novel cathelicidin gene in the rainbow trout, *Oncorhynchus mykiss*. Infect. Immun..

[28] Lu X.J., Chen J., Huang Z.A., Shi Y.H., Lv J.N. (2011). Identification and characterization of a novel cathelicidin from ayu, *Plecoglossus altivelis*. Fish Shellfish Immunol..

[29] Li Z., Zhang S., Gao J., Guang H., Tian Y., Zhao Z., Wang Y., Yu H. (2013). Structural and functional characterization of CATH_BRALE, the defense molecule in the ancient salmonoid, *Brachymystax lenok*. Fish Shellfish Immunol..

[30] Maier V.H., Schmitt C.N., Gudmundsdottir S., Gudmundsson G.H. (2008). Bacterial DNA indicated as an important inducer of fish cathelicidins. Mol. Immunol..

[31] Bridle A., Nosworthy E., Polinski M., Nowak B. (2011). Evidence of an antimicrobial-immunomodulatory role of Atlantic salmon cathelicidins during infection with *Yersinia ruckeri*. PLoS ONE.

[32] Chang C.I., Zhang Y.A., Zou J., Nie P., Secombes C.J. (2006). Two cathelicidin genes are present in both rainbow trout (*Oncorhynchus mykiss*) and atlantic salmon (*Salmo salar*). Antimicrob. Agents Chemother..

[33] Zhang X.J., Zhang X.Y., Zhang N., Guo X., Peng K.S., Wu H., Lu L.F., Wu N., Chen D.D., Li S. (2015). Distinctive structural hallmarks and biological activities of the multiple cathelicidin antimicrobial peptides in a primitive teleost fish. J. Immunol..

[34] Broekman D.C., Frei D.M., Gylfason G.A., Steinarsson A., Jornvall H., Agerberth B., Gudmundsson G.H., Maier V.H. (2011). Cod cathelicidin: Isolation of the mature peptide, cleavage site characterisation and developmental expression. Dev. Comp. Immunol..

[35] Broekman D.C., Zenz A., Gudmundsdottir B.K., Lohner K., Maier V.H., Gudmundsson G.H. (2011). Functional characterization of codCath, the mature cathelicidin antimicrobial peptide from Atlantic cod (*Gadus morhua*). Peptides.

[36] Broekman D.C., Guethmundsson G.H., Maier V.H. (2013). Differential regulation of cathelicidin in salmon and cod. Fish Shellfish Immunol..

[37] Shewring D.M., Zou J., Corripio-Miyar Y., Secombes C.J. (2011). Analysis of the cathelicidin 1 gene locus in Atlantic cod (*Gadus morhua*). Mol. Immunol..

[38] Feng C.Y., Johnson S.C., Hori T.S., Rise M., Hall J.R., Gamperl A.K., Hubert S., Kimball J., Bowman S., Rise M.L. (2009). Identification and analysis of differentially expressed genes in immune tissues of Atlantic cod stimulated with formalin-killed, atypical *Aeromonas salmonicida*. Physiol. Genomics.

[39] De Bruijn I., Belmonte R., Anderson V.L., Saraiva M., Wang T., van West P., Secombes C.J. (2012). Immune gene expression in trout cell lines infected with the fish pathogenic oomycete *Saprolegnia parasitica*. Dev. Comp. Immunol..

[40] Costa M.M., Maehr T., Diaz-Rosales P., Secombes C.J., Wang T. (2011). Bioactivity studies of rainbow trout (*Oncorhynchus mykiss*) interleukin-6: Effects on macrophage growth and antimicrobial peptide gene expression. Mol. Immunol..

[41] Schmitt P., Wacyk J., Morales-Lange B., Rojas V., Guzman F., Dixon B., Mercado L. (2015). Immunomodulatory effect of cathelicidins in response to a beta-glucan in intestinal epithelial cells from rainbow trout. Dev. Comp. Immunol..

[42] Krasnov A., Wesmajervi Breiland M.S., Hatlen B., Afanasyev S., Skugor S. (2015). Sexual maturation and administration of 17β-estradiol and testosterone induce complex gene expression changes in skin and increase resistance of Atlantic salmon to ectoparasite salmon louse. Gen. Comp. Endocrinol..

[43] Sun B.J., Xie H.X., Song Y., Nie P. (2007). Gene structure of an antimicrobial peptide from mandarin fish, *Siniperca chuatsi* (Basilewsky), suggests that moronecidins and pleurocidins belong in one family: The piscidins. J. Fish Dis..

[44] Pan C.Y., Chen J.Y., Ni I.H., Wu J.L., Kuo C.M. (2008). Organization and promoter analysis of the grouper (*Epinephelus coioides*) epinecidin-1 gene. Comp. Biochem. Physiol. B Biochem. Mol. Biol..

[45] Browne M.J., Feng C.Y., Booth V., Rise M.L. (2011). Characterization and expression studies of Gaduscidin-1 and Gaduscidin-2; paralogous antimicrobial peptide-like transcripts from Atlantic cod (*Gadus morhua*). Dev. Comp. Immunol..

[46] Dezfuli B.S., Pironi F., Giari L., Noga E.J. (2010). Immunocytochemical localization of piscidin in mast cells of infected seabass gill. Fish Shellfish Immunol..

[47] Buonocore F., Randelli E., Casani D., Picchietti S., Belardinelli M.C., de Pascale D., De Santi C., Scapigliati G. (2012). A piscidin-like antimicrobial peptide from the icefish *Chionodraco hamatus* (Perciformes: Channichthyidae): Molecular characterization, localization and bactericidal activity. Fish Shellfish Immunol..

[48] Bae J.S., Shim S.H., Hwang S.D., Park M.A., Jee B.Y., An C.M., Kim Y.O., Kim J.W., Park C.I. (2014). Expression analysis and biological activity of moronecidin from rock bream, *Oplegnathus fasciatus*. Fish Shellfish Immunol..

[49] Niu S.F., Jin Y., Xu X., Qiao Y., Wu Y., Mao Y., Su Y.Q., Wang J. (2013). Characterization of a novel piscidin-like antimicrobial peptide from *Pseudosciaena crocea* and its immune response to *Cryptocaryon irritans*. Fish Shellfish Immunol..

[50] Zhou Q.J., Su Y.Q., Niu S.F., Liu M., Qiao Y., Wang J. (2014). Discovery and molecular cloning of piscidin-5-like gene from the large yellow croaker (*Larimichthys crocea*). Fish Shellfish Immunol..

[51] Andrews M., Battaglene S., Cobcroft J., Adams M., Noga E., Nowak B. (2010). Host response to the chondracanthid copepod *Chondracanthus goldsmidi*, a gill parasite of the striped trumpeter, Latris lineata (Forster), in Tasmania. J. Fish Dis..

[52] Patrzykat A., Gallant J.W., Seo J.K., Pytyck J., Douglas S.E. (2003). Novel antimicrobial peptides derived from flatfish genes. Antimicrob. Agents Chemother..

[53] Fernandes J.M., Ruangsri J., Kiron V. (2010). Atlantic cod piscidin and its diversification through positive selection. PLoS ONE.

[54] Ruangsri J., Salger S.A., Caipang C.M., Kiron V., Fernandes J.M. (2012). Differential expression and biological activity of two piscidin paralogues and a novel splice variant in Atlantic cod (*Gadus morhua* L.). Fish Shellfish Immunol..

[55] Lauth X., Shike H., Burns J.C., Westerman M.E., Ostland V.E., Carlberg J.M., Van Olst J.C., Nizet V., Taylor S.W., Shimizu C., Bulet P. (2002). Discovery and characterization of two isoforms of moronecidin, a novel antimicrobial peptide from hybrid striped bass. J. Biol. Chem..

[56] Noga E.J., Silphaduang U., Park N.G., Seo J.K., Stephenson J., Kozlowicz S. (2009). Piscidin 4, a novel member of the piscidin family of antimicrobial peptides. Comp. Biochem. Physiol. B Biochem. Mol. Biol..

[57] Salger S.A., Reading B.J., Baltzegar D.A., Sullivan C.V., Noga E.J. (2011). Molecular characterization of two isoforms of piscidin 4 from the hybrid striped bass (*Morone chrysops* x *Morone saxatilis*). Fish Shellfish Immunol..

[58] Douglas S.E., Patrzykat A., Pytyck J., Gallant J.W. (2003). Identification, structure and differential expression of novel pleurocidins clustered on the genome of the winter flounder, *Pseudopleuronectes americanus* (Walbaum). Eur. J. Biochem..

[59] Douglas S.E., Gallant J.W., Gong Z., Hew C. (2001). Cloning and developmental expression of a family of pleurocidin-like antimicrobial peptides from winter flounder, *Pleuronectes americanus* (Walbaum). Dev. Comp. Immunol..

[60] Peng K.C., Lee S.H., Hour A.L., Pan C.Y., Lee L.H., Chen J.Y. (2012). Five different piscidins from *Nile tilapia*, *Oreochromis niloticus*: Analysis of their expressions and biological functions. PLoS ONE.

[61] Cole A.M., Darouiche R.O., Legarda D., Connell N., Diamond G. (2000). Characterization of a fish antimicrobial peptide: gene expression, subcellular localization, and spectrum of activity. Antimicrob. Agents Chemother..

[62] Cole A.M., Weis P., Diamond G. (1997). Isolation and characterization of pleurocidin, an antimicrobial peptide in the skin secretions of winter flounder. J. Biol. Chem..

[63] Syvitski R.T., Burton I., Mattatall N.R., Douglas S.E., Jakeman D.L. (2005). Structural characterization of the antimicrobial peptide pleurocidin from winter flounder. Biochemistry.

[64] Chekmenev E.Y., Vollmar B.S., Forseth K.T., Manion M.N., Jones S.M., Wagner T.J., Endicott R.M., Kyriss B.P., Homem L.M., Pate M. (2006). Investigating molecular recognition and biological function at interfaces using piscidins, antimicrobial peptides from fish. Biochim. Biophys. Acta.

[65] Tennessen J.A. (2005). Enhanced synonymous site divergence in positively selected vertebrate antimicrobial peptide genes. J. Mol. Evol..

[66] Pan C.Y., Chen J.Y., Cheng Y.S., Chen C.Y., Ni I.H., Sheen J.F., Pan Y.L., Kuo C.M. (2007). Gene expression and localization of the epinecidin-1 antimicrobial peptide in the grouper (*Epinephelus coioides*), and its role in protecting fish against pathogenic infection. DNA Cell Biol..

[67] Ruangsri J., Fernandes J.M., Rombout J.H., Brinchmann M.F., Kiron V. (2012). Ubiquitous presence of piscidin-1 in Atlantic cod as evidenced by immunolocalisation. BMC Vet. Res..

[68] Corrales J., Mulero I., Mulero V., Noga E.J. (2010). Detection of antimicrobial peptides related to piscidin 4 in important aquacultured fish. Dev. Comp. Immunol..

[69] Corrales J., Gordon W.L., Noga E.J. (2009). Development of an ELISA for quantification of the antimicrobial peptide piscidin 4 and its application to assess stress in fish. Fish Shellfish Immunol..

[70] Silphaduang U., Colorni A., Noga E.J. (2006). Evidence for widespread distribution of piscidin antimicrobial peptides in teleost fish. Dis. Aquat. Organ..

[71] Murray H.M., Gallant J.W., Douglas S.E. (2003). Cellular localization of pleurocidin gene expression and synthesis in winter flounder gill using immunohistochemistry and *in situ* hybridization. Cell Tissue Res..

[72] Mulero I., Noga E.J., Meseguer J., Garcia-Ayala A., Mulero V. (2008). The antimicrobial peptides piscidins are stored in the granules of professional phagocytic granulocytes of fish and are delivered to the bacteria-containing phagosome upon phagocytosis. Dev. Comp. Immunol..

[73] Peter Chiou P., Khoo J., Bols N.C., Douglas S., Chen T.T. (2006). Effects of linear cationic alpha-helical antimicrobial peptides on immune-relevant genes in trout macrophages. Dev. Comp. Immunol..

[74] Chinchar V.G., Bryan L., Silphadaung U., Noga E., Wade D., Rollins-Smith L. (2004). Inactivation of viruses infecting ectothermic animals by amphibian and piscine antimicrobial peptides. Virology.

[75] Sung W.S., Lee J., Lee D.G. (2008). Fungicidal effect and the mode of action of piscidin 2 derived from hybrid striped bass. Biochem. Biophys. Res. Commun..

[76] Sung W.S., Lee J., Lee D.G. (2008). Fungicidal effect of piscidin on *Candida albicans*: Pore formation in lipid vesicles and activity in fungal membranes. Biol. Pharm. Bull..

[77] Colorni A., Ullal A., Heinisch G., Noga E.J. (2008). Activity of the antimicrobial polypeptide piscidin 2 against fish ectoparasites. J. Fish Dis..

[78] Park N.G., Silphaduang U., Moon H.S., Seo J.K., Corrales J., Noga E.J. (2011). Structure-activity relationships of piscidin 4, a piscine antimicrobial peptide. Biochemistry.

[79] Dezfuli B.S., Lui A., Boldrini P., Pironi F., Giari L. (2008). The inflammatory response of fish to helminth parasites. Parasite.

[80] Morash M.G., Douglas S.E., Robotham A., Ridley C.M., Gallant J.W., Soanes K.H. (2011). The zebrafish embryo as a tool for screening and characterizing pleurocidin host-defense peptides as anti-cancer agents. Dis. Model. Mech..

[81] Hilchie A.L., Doucette C.D., Pinto D.M., Patrzykat A., Douglas S., Hoskin D.W. (2011). Pleurocidin-family cationic antimicrobial peptides are cytolytic for breast carcinoma cells and prevent growth of tumor xenografts. Breast Cancer Res..

[82] Lin H.J., Huang T.C., Muthusamy S., Lee J.F., Duann Y.F., Lin C.H. (2012). Piscidin-1, an antimicrobial peptide from fish (hybrid striped bass *Morone saxatilis* x *M. chrysops*), induces apoptotic and necrotic activity in HT1080 cells. Zoolog. Sci..

[83] Chen J.Y., Lin W.J., Wu J.L., Her G.M., Hui C.F. (2009). Epinecidin-1 peptide induces apoptosis which enhances antitumor effects in human leukemia U937 cells. Peptides.

[84] Wang Y.D., Kung C.W., Chi S.C., Chen J.Y. (2010). Inactivation of nervous necrosis virus infecting grouper (*Epinephelus coioides*) by epinecidin-1 and hepcidin 1–5 antimicrobial peptides, and downregulation of Mx2 and Mx3 gene expressions. Fish Shellfish Immunol..

[85] Patrzykat A., Zhang L., Mendoza V., Iwama G.K., Hancock R.E. (2001). Synergy of histone-derived peptides of coho salmon with lysozyme and flounder pleurocidin. Antimicrob. Agents Chemother..

[86] Yoshida K., Mukai Y., Niidome T., Takashi C., Tokunaga Y., Hatakeyama T., Aoyagi H. (2001). Interaction of pleurocidin and its analogs with phospholipid membrane and their antibacterial activity. J. Pept. Res..

[87] Campagna S., Saint N., Molle G., Aumelas A. (2007). Structure and mechanism of action of the antimicrobial peptide piscidin. Biochemistry.

[88] Saint N., Cadiou H., Bessin Y., Molle G. (2002). Antibacterial peptide pleurocidin forms ion channels in planar lipid bilayers. Biochim. Biophys. Acta.

[89] Cho J., Lee D.G. (2011). Oxidative stress by antimicrobial peptide pleurocidin triggers apoptosis in Candida albicans. Biochimie.

[90] Meloni M., Candusso S., Galeotti M., Volpatti D. (2015). Preliminary study on expression of antimicrobial peptides in European sea bass (*Dicentrarchus labrax*) following *in vivo* infection with *Vibrio anguillarum*. A time course experiment. Fish Shellfish Immunol..

[91] Dezfuli B.S., Manera M., Lorenzoni M., Pironi F., Shinn A.P., Giari L. (2015). Histopathology and the inflammatory response of European perch, *Perca fluviatilis* muscle infected with *Eustrongylides* sp. (Nematoda). Parasites Vectors.

[92] Dezfuli B.S., Lui A., Giari L., Pironi F., Manera M., Lorenzoni M., Noga E.J. (2013). Piscidins in the intestine of European perch, *Perca fluviatilis*, naturally infected with an enteric worm. Fish Shellfish Immunol..

[93] Dezfuli B.S., Lui A., Giari L., Castaldelli G., Mulero V., Noga E.J. (2012). Infiltration and activation of acidophilic granulocytes in skin lesions of *gilthead seabream*, *Sparus aurata*, naturally infected with lymphocystis disease virus. Dev. Comp. Immunol..

[94] Dezfuli B.S., Giari L., Lui A., Lorenzoni M., Noga E.J. (2011). Mast cell responses to *Ergasilus* (Copepoda), a gill ectoparasite of sea bream. Fish Shellfish Immunol..

[95] Pan C.Y., Wu J.L., Hui C.F., Lin C.H., Chen J.Y. (2011). Insights into the antibacterial and immunomodulatory functions of the antimicrobial peptide, epinecidin-1, against *Vibrio vulnificus* infection in zebrafish. Fish Shellfish Immunol..

[96] Pan C.Y., Huang T.C., Wang Y.D., Yeh Y.C., Hui C.F., Chen J.Y. (2012). Oral administration of recombinant epinecidin-1 protected grouper (*Epinephelus coioides*) and zebrafish (*Danio rerio*) from *Vibrio vulnificus* infection and enhanced immune-related gene expressions. Fish Shellfish Immunol..

[97] Lee L.H., Hui C.F., Chuang C.M., Chen J.Y. (2013). Electrotransfer of the epinecidin-1 gene into skeletal muscle enhances the antibacterial and immunomodulatory functions of a marine fish, grouper (*Epinephelus coioides*). Fish Shellfish Immunol..

[98] Peng K.C., Pan C.Y., Chou H.N., Chen J.Y. (2010). Using an improved Tol2 transposon system to produce transgenic zebrafish with epinecidin-1 which enhanced resistance to bacterial infection. Fish Shellfish Immunol..

[99] Wang Y.D., Kung C.W., Chen J.Y. (2010). Antiviral activity by fish antimicrobial peptides of epinecidin-1 and hepcidin 1–5 against nervous necrosis virus in medaka. Peptides.

[100] Wang Y.D., Rajanbabu V., Chen J.Y. (2015). Transcriptome analysis of medaka following epinecidin-1 and TH1–5 treatment of NNV infection. Fish Shellfish Immunol..

[101] Huang T.C., Chen J.Y. (2013). Proteomic and functional analysis of zebrafish after administration of antimicrobial peptide epinecidin-1. Fish Shellfish Immunol..

[102] Krause A., Neitz S., Magert H.J., Schulz A., Forssmann W.G., Schulz-Knappe P., Adermann K. (2000). LEAP-1, a novel highly disulfide-bonded human peptide, exhibits antimicrobial activity. FEBS Lett..

[103] Park C.H., Valore E.V., Waring A.J., Ganz T. (2001). Hepcidin, a urinary antimicrobial peptide synthesized in the liver. J. Biol. Chem..

[104] Shi J., Camus A.C. (2006). Hepcidins in amphibians and fishes: Antimicrobial peptides or iron-regulatory hormones?. Dev. Comp. Immunol..

[105] Wang K.J., Cai J.J., Cai L., Qu H.D., Yang M., Zhang M. (2009). Cloning and expression of a hepcidin gene from a marine fish (*Pseudosciaena crocea*) and the antimicrobial activity of its synthetic peptide. Peptides.

[106] Bao B.L., Peatman E., Li P., He C.B., Liu Z.J. (2005). Catfish hepcidin gene is expressed in a wide range of tissues and exhibits tissue-specific upregulation after bacterial infection. Dev. Comp. Immunol..

[107] Bao B., Peatman E., Xu P., Li P., Zeng H., He C., Liu Z. (2006). The catfish liver-expressed antimicrobial peptide 2 (LEAP-2) gene is expressed in a wide range of tissues and developmentally regulated. Mol. Immunol..

[108] Shike H., Lauth X., Westerman M.E., Ostland V.E., Carlberg J.M., Van Olst J.C., Shimizu C., Bulet P., Burns J.C. (2002). Bass hepcidin is a novel antimicrobial peptide induced by bacterial challenge. Eur. J. Biochem..

[109] Shike H., Shimizu C., Lauth X., Burns J.C. (2004). Organization and expression analysis of the zebrafish hepcidin gene, an antimicrobial peptide gene conserved among vertebrates. Dev. Comp. Immunol..

[110] Cai L., Cai J.J., Liu H.P., Fan D.Q., Peng H., Wang K.J. (2012). Recombinant medaka (*Oryzias melastigmus*) pro-hepcidin: Multifunctional characterization. Comp. Biochem. Phys. B Biochem. Mol. Biol..

[111] Zhang J., Yu L.P., Li M.F., Sun L. (2014). Turbot (*Scophthalmus maximus*) hepcidin-1 and hepcidin-2 possess antimicrobial activity and promote resistance against bacterial and viral infection. Fish Shellfish Immunol..

[112] Douglas S.E., Gallant J.W., Liebscher R.S., Dacanay A., Tsoi S.C. (2003). Identification and expression analysis of hepcidin-like antimicrobial peptides in bony fish. Dev. Comp. Immunol..

[113] Zhang Y.A., Zou J., Chang C.I., Secombes C.J. (2004). Discovery and characterization of two types of liver-expressed antimicrobial peptide 2 (LEAP-2) genes in rainbow trout. Vet. Immunol. Immunopathol..

[114] Kim Y.O., Hong S., Nam B.H., Lee J.H., Kim K.K., Lee S.J. (2005). Molecular cloning and expression analysis of two hepcidin genes from olive flounder *Paralichthys olivaceus*. Biosci. Biotechnol. Biochem..

[115] Kim Y.O., Park E.M., Nam B.H., Kong H.J., Kim W.J., Lee S.J. (2008). Identification and molecular characterization of two hepcidin genes from black rockfish (*Sebastes schlegelii*). Mol. Cell. Biochem..

[116] Barnes A.C., Trewin B., Snape N., Kvennefors E.C., Baiano J.C. (2011). Two hepcidin-like antimicrobial peptides in Barramundi *Lates calcarifer* exhibit differing tissue tropism and are induced in response to lipopolysaccharide. Fish Shellfish Immunol..

[117] Huang P.H., Chen J.Y., Kuo C.M. (2007). Three different hepcidins from tilapia, *Oreochromis mossambicus*: Analysis of their expressions and biological functions. Mol. Immunol..

[118] Qu H.D., Chen B., Peng H., Wang K.J. (2013). Molecular cloning, recombinant expression, and antimicrobial activity of EC-hepcidin3, a new four-cysteine hepcidin isoform from Epinephelus coioides. Biosci. Biotech. Biochem..

[119] Zhou J.G., Wei J.G., Xu D., Cui H.C., Yan Y., Ou-Yang Z.L., Huang X.H., Huang Y.H., Qin Q.W. (2011). Molecular cloning and characterization of two novel hepcidins from orange-spotted grouper, *Epinephelus coioides*. Fish Shellfish Immunol..

[120] Cho Y.S., Lee S.Y., Kim K.H., Kim S.K., Kim D.S., Nam Y.K. (2009). Gene structure and differential modulation of multiple rockbream (*Oplegnathus fasciatus*) hepcidin isoforms resulting from different biological stimulations. Dev. Comp. Immunol..

[121] Zheng W., Liu G., Ao J., Chen X. (2006). Expression analysis of immune-relevant genes in the spleen of large yellow croaker (*Pseudosciaena crocea*) stimulated with poly I:C. Fish Shellfish Immunol..

[122] Yang M., Wang K.J., Chen J.H., Qu H.D., Li S.J. (2007). Genomic organization and tissue-specific expression analysis of hepcidin-like genes from black porgy (*Acanthopagrus schlegelii* B). Fish Shellfish Immunol..

[123] Chen S.L., Xu M.Y., Ji X.S., Yu G.C., Liu Y. (2005). Cloning, characterization, and expression analysis of hepcidin gene from red sea bream (*Chrysophrys major*). Antimicrob. Agents Chemother..

[124] Chen S.L., Li W., Meng L., Sha Z.X., Wang Z.J., Ren G.C. (2007). Molecular cloning and expression analysis of a hepcidin antimicrobial peptide gene from turbot (*Scophthalmus maximus*). Fish Shellfish Immunol..

[125] Srinivasulu B., Syvitski R., Seo J.K., Mattatall N.R., Knickle L.C., Douglas S.E. (2008). Expression, purification and structural characterization of recombinant hepcidin, an antimicrobial peptide identified in Japanese flounder, Paralichthys olivaceus. Protein Expr. Purif..

[126] Lin W., Liu S., Hu L., Zhang S. (2014). Characterization and bioactivity of hepcidin-2 in zebrafish: Dependence of antibacterial activity upon disulfide bridges. Peptides.

[127] Mao M.G., Jiang J.L., Peralvarez-Marin A., Wang K.J., Lei J.L. (2013). Characterization of the Mx and hepcidin genes in Epinephelus akaara asymptomatic carriers of the nervous necrosis virus. Aquaculture.

[128] Cuesta A., Meseguer J., Esteban M.A. (2008). The antimicrobial peptide hepcidin exerts an important role in the innate immunity against bacteria in the bony fish gilthead seabream. Mol. Immunol..

[129] Martin-Antonio B., Jimenez-Cantizano R.M., Salas-Leiton E., Infante C., Manchado M. (2009). Genomic characterization and gene expression analysis of four hepcidin genes in the redbanded seabream (*Pagrus auriga*). Fish Shellfish Immunol..

[130] Yang M., Chen B., Cai J.J., Peng H., Ling C., Yuan J.J., Wang K.J. (2011). Molecular characterization of hepcidin AS-hepc2 and AS-hepc6 in black porgy (*Acanthopagrus schlegelii*): expression pattern responded to bacterial challenge and *in vitro* antimicrobial activity. Comp. Biochem. Physiol. B Biochem. Mol. Biol..

[131] Yang G., Guo H., Li H., Shan S., Zhang X., Rombout J.H., An L. (2014). Molecular characterization of LEAP-2 cDNA in common carp (*Cyprinus carpio* L.) and the differential expression upon a *Vibrio anguillarum* stimulus; indications for a significant immune role in skin. Fish Shellfish Immunol..

[132] Neves J.V., Caldas C., Vieira I., Ramos M.F., Rodrigues P.N. (2015). Multiple Hepcidins in a Teleost Fish, Dicentrarchus labrax: Different Hepcidins for Different Roles. J. Immunol..

[133] Hirono I., Hwang J.Y., Ono Y., Kurobe T., Ohira T., Nozaki R., Aoki T. (2005). Two different types of hepcidins from the Japanese flounder *Paralichthys olivaceus*. FEBS J..

[134] Pereiro P., Figueras A., Novoa B. (2012). A novel hepcidin-like in turbot (*Scophthalmus maximus* L.) highly expressed after pathogen challenge but not after iron overload. Fish Shellfish Immunol..

[135] Hu X., Camus A.C., Aono S., Morrison E.E., Dennis J., Nusbaum K.E., Judd R.L., Shi J. (2007). Channel catfish hepcidin expression in infection and anemia. Comp. Immunol. Microbiol. Infect. Dis..

[136] Padhi A., Verghese B. (2007). Evidence for positive Darwinian selection on the hepcidin gene of Perciform and Pleuronectiform fishes. Mol. Divers..

[137] Xu Q., Cheng C.H., Hu P., Ye H., Chen Z., Cao L., Chen L., Shen Y., Chen L. (2008). Adaptive evolution of hepcidin genes in antarctic notothenioid fishes. Mol. Biol. Evol..

[138] Tao Y., Zhao D.M., Wen Y. (2014). Expression, purification and antibacterial activity of the channel catfish hepcidin mature peptide. Protein Expr. Purif..

[139] Alvarez C.A., Guzman F., Cardenas C., Marshall S.H., Mercado L. (2014). Antimicrobial activity of trout hepcidin. Fish Shellfish Immunol..

[140] Pan C.Y., Peng K.C., Lin C.H., Chen J.Y. (2011). Transgenic expression of tilapia hepcidin 1–5 and shrimp chelonianin in zebrafish and their resistance to bacterial pathogens. Fish Shellfish Immunol..

[141] Hsieh J.C., Pan C.Y., Chen J.Y. (2010). Tilapia hepcidin (TH)2–3 as a transgene in transgenic fish enhances resistance to *Vibrio vulnificus* infection and causes variations in immune-related genes after infection by different bacterial species. Fish Shellfish Immunol..

[142] Lauth X., Babon J.J., Stannard J.A., Singh S., Nizet V., Carlberg J.M., Ostland V.E., Pennington M.W., Norton R.S., Westerman M.E. (2005). Bass hepcidin synthesis, solution structure, antimicrobial activities and synergism, and *in vivo* hepatic response to bacterial infections. J. Biol. Chem..

[143] Chen J., Shi Y.H., Li M.Y. (2008). Changes in transferrin and hepcidin genes expression in the liver of the fish Pseudosciaena crocea following exposure to cadmium. Arch. Toxicol..

[144] Fraenkel P.G., Gibert Y., Holzheimer J.L., Lattanzi V.J., Burnett S.F., Dooley K.A., Wingert R.A., Zon L.I. (2009). Transferrin-a modulates hepcidin expression in zebrafish embryos. Blood.

[145] Steinbicker A.U., Sachidanandan C., Vonner A.J., Yusuf R.Z., Deng D.Y., Lai C.S., Rauwerdink K.M., Winn J.C., Saez B., Cook C.M., Szekely B.A. (2011). Inhibition of bone morphogenetic protein signaling attenuates anemia associated with inflammation. Blood.

[146] Gibert Y., Lattanzi V.J., Zhen A.W., Vedder L., Brunet F., Faasse S.A., Babitt J.L., Lin H.Y., Hammerschmidt M., Fraenkel P.G. (2011). BMP signaling modulates hepcidin expression in zebrafish embryos independent of hemojuvelin. PLoS ONE.

[147] Neves J.V., Caldas C., Wilson J.M., Rodrigues P.N. (2011). Molecular mechanisms of hepcidin regulation in sea bass (*Dicentrarchus labrax*). Fish Shellfish Immunol..

[148] Lin B., Chen S., Cao Z., Lin Y., Mo D., Zhang H., Gu J., Dong M., Liu Z., Xu A. (2007). Acute phase response in zebrafish upon Aeromonas salmonicida and Staphylococcus aureus infection: Striking similarities and obvious differences with mammals. Mol. Immunol..

[149] Gerwick L., Corley-Smith G., Bayne C.J. (2007). Gene transcript changes in individual rainbow trout livers following an inflammatory stimulus. Fish Shellfish Immunol..

[150] Wang K.J., Bo J., Yang M., Hong H.S., Wang X.H., Chen F.Y., Yuan J.J. (2009). Hepcidin gene expression induced in the developmental stages of fish upon exposure to Benzo[a]pyrene (BaP). Mar. Environ. Res..

[151] Straub P.F., Higham M.L., Tanguy A., Landau B.J., Phoel W.C., Hales L.S., Thwing T.K. (2004). Suppression subtractive hybridization cDNA libraries to identify differentially expressed genes from contrasting fish habitats. Mar. Biotechnol..

[152] Huang Z.H., Ma A.J., Wang X.A. (2011). The immune response of turbot, *Scophthalmus maximus* (L.), skin to high water temperature. J. Fish Dis..

[153] Rajanbabu V., Chen J.Y. (2011). Antiviral function of tilapia hepcidin 1–5 and its modulation of immune-related gene expressions against infectious pancreatic necrosis virus (IPNV) in Chinook salmon embryo (CHSE)-214 cells. Fish Shellfish Immunol..

[154] Rajanbabu V., Chen J.Y. (2011). The antimicrobial peptide, tilapia hepcidin 2–3, and PMA differentially regulate the protein kinase C isoforms, TNF-alpha and COX-2, in mouse RAW264.7 macrophages. Peptides.

[155] Fernandes J.M., Molle G., Kemp G.D., Smith V.J. (2004). Isolation and characterisation of oncorhyncin II, a histone H1-derived antimicrobial peptide from skin secretions of rainbow trout, *Oncorhynchus mykiss*. Dev. Comp. Immunol..

[156] Luders T., Birkemo G.A., Nissen-Meyer J., Andersen O., Nes I.F. (2005). Proline conformation-dependent antimicrobial activity of a proline-rich histone h1 N-terminal Peptide fragment isolated from the skin mucus of Atlantic salmon. Antimicrob. Agents Chemother..

[157] Shamova O.V., Orlov D.S., Balandin S.V., Shramova E.I., Tsvetkova E.V., Panteleev P.V., Leonova Y.F., Tagaev A.A., Kokryakov V.N., Ovchinnikova T.V. (2014). Acipensins—Novel antimicrobial peptides from Leukocytes of the Russian Sturgeon *Acipenser gueldenstaedtii*. Acta Naturae.

[158] Birkemo G.A., Luders T., Andersen O., Nes I.F., Nissen-Meyer J. (2003). Hipposin, a histone-derived antimicrobial peptide in Atlantic halibut (*Hippoglossus hippoglossus* L.). Biochim. Biophys. Acta.

[159] Park I.Y., Park C.B., Kim M.S., Kim S.C. (1998). Parasin I, an antimicrobial peptide derived from histone H2A in the catfish, *Parasilurus asotus*. FEBS Lett..

[160] Noga E.J., Fan Z.Q., Silphaduang U. (2002). Host site of activity and cytological effects of histone-like proteins on the parasitic dinoflagellate *Amyloodinium ocellatum*. Dis. Aquat. Organ..

[161] Bustillo M.E., Fischer A.L., LaBouyer M.A., Klaips J.A., Webb A.C., Elmore D.E. (2014). Modular analysis of hipposin, a histone-derived antimicrobial peptide consisting of membrane translocating and membrane permeabilizing fragments. Biochim. Biophys. Acta.

[162] Caipang C.M., Lazado C.C., Brinchmann M.F., Kiron V. (2010). Infection-induced changes in expression of antibacterial and cytokine genes in the gill epithelial cells of Atlantic cod, *Gadus morhua* during incubation with bacterial pathogens. Comp. Biochem. Physiol. B Biochem. Mol. Biol..

[163] Martin S.A., Blaney S.C., Houlihan D.F., Secombes C.J. (2006). Transcriptome response following administration of a live bacterial vaccine in Atlantic salmon (*Salmo salar*). Mol. Immunol..

[164] Santana P.A., Alvarez C.A., Guzman F., Mercado L. (2013). Development of a sandwich ELISA for quantifying hepcidin in Rainbow trout. Fish Shellfish Immunol..

[165] Chiou P.P., Lin C.M., Bols N.C., Chen T.T. (2007). Characterization of virus/double-stranded RNA-dependent induction of antimicrobial peptide hepcidin in trout macrophages. Dev. Comp. Immunol..

[166] Matsuyama T., Fujiwara A., Nakayasu C., Kamaishi T., Oseko N., Hirono I., Aoki T. (2007). Gene expression of leucocytes in vaccinated Japanese flounder (*Paralichthys olivaceus*) during the course of experimental infection with *Edwardsiella tarda*. Fish Shellfish Immunol..

[167] Alvarez C.A., Santana P.A., Guzman F., Marshall S., Mercado L. (2013). Detection of the hepcidin prepropeptide and mature peptide in liver of rainbow trout. Dev. Comp. Immunol..

[168] Chi J.R., Liao L.S., Wang R.G., Jhu C.S., Wu J.L., Hu S.Y. (2015). Molecular cloning and functional characterization of the hepcidin gene from the convict cichlid (*Amatitlania nigrofasciata*) and its expression pattern in response to lipopolysaccharide challenge. Fish Physiol. Biochem..

[169] Li H., Zhang F., Guo H., Zhu Y., Yuan J., Yang G., An L. (2013). Molecular characterization of hepcidin gene in common carp (*Cyprinus carpio* L.) and its expression pattern responding to bacterial challenge. Fish Shellfish Immunol..

[170] Chettri J.K., Raida M.K., Kania P.W., Buchmann K. (2012). Differential immune response of rainbow trout (*Oncorhynchus mykiss*) at early developmental stages (larvae and fry) against the bacterial pathogen *Yersinia ruckeri*. Dev. Comp. Immunol..

[171] Valero Y., Garcia-Alcazar A., Esteban M.A., Cuesta A., Chaves-Pozo E. (2015). Antimicrobial response is increased in the testis of European sea bass, but not in gilthead seabream, upon nodavirus infection. Fish Shellfish Immunol..

[172] Mohd-Shaharuddin N., Mohd-Adnan A., Kua B.C., Nathan S. (2013). Expression profile of immune-related genes in *Lates calcarifer* infected by *Cryptocaryon irritans*. Fish Shellfish Immunol..

[173] Chettri J.K., Kuhn J.A., Jaafar R.M., Kania P.W., Moller O.S., Buchmann K. (2014). Epidermal response of rainbow trout to Ichthyobodo necator: Immunohistochemical and gene expression studies indicate a Th1-/Th2-like switch. J. Fish Dis..

[174] Heinecke R.D., Buchmann K. (2013). Inflammatory response of rainbow trout *Oncorhynchus mykiss* (Walbaum, 1792) larvae against Ichthyophthirius multifiliis. Fish Shellfish Immunol..

[175] Noga E.J., Ullal A.J., Corrales J., Fernandes J.M. (2011). Application of antimicrobial polypeptide host defenses to aquaculture: Exploitation of downregulation and upregulation responses. Comp. Biochem. Physiol. Part D Genomics Proteomics.

[176] Lombardi L., Maisetta G., Batoni G., Tavanti A. (2015). Insights into the antimicrobial properties of hepcidins: Advantages and drawbacks as potential therapeutic agents. Molecules.

[177] Brown K.L., Hancock R.E. (2006). Cationic host defense (antimicrobial) peptides. Curr. Opin. Immunol..

[178] Rakers S., Niklasson L., Steinhagen D., Kruse C., Schauber J., Sundell K., Paus R. (2013). Antimicrobial peptides (AMPs) from fish epidermis: Perspectives for investigative dermatology. J. Invest. Dermatol..

